# Supplementary material for: Mitochondrial DNA variant detection in over 6,500 rare disease families by the systematic analysis of exome and genome sequencing data resolves undiagnosed cases
Source: HGG Adv. 2025 Apr 15;6(3):100441. doi: 10.1016/j.xhgg.2025.100441 (PMC12278631; doi:10.1016/j.xhgg.2025.100441)
Supplement: Document S2. Article plus supplemental information [file mmc3.pdf]

HGG
Advances

ARTICLE

Mitochondrial DNA variant detection in over 6,500 rare disease families by the systematic analysis of exome and genome sequencing data resolves undiagnosed cases

Sarah L. Stenton,[1,2] Kristen Laricchia,[1] Nicole J. Lake,[3] Sushma Chaluvadi,[1] Vijay Ganesh,[1] Stephanie DiTroia,[1] Ikeoluwa Osei-Owusu,[1] Lynn Pais,[1] Emily O'Heir,[1] Christina Austin-Tse,[1,4] Melanie O'Leary,[1] Mayada Abu Shanap,[5] Chelsea Barrows,[6,7] Seth Berger,[8] Carsten G. Bönnemann,[9] Kinga M. Bujakowska,[10] Dean R. Campagna,[11] Alison G. Compton,[12,13] Sandra Donkervoort,[9] Mark D. Fleming,[11] Lyndon Gallacher,[12,13] Joseph G. Gleeson,[6,7] Goknur Haliloglu,[9] Eric A. Pierce,[10] Emily M. Place,[10] Vijay G. Sankaran,[14,15,16,17] Akiko Shimamura,[14,15] Zornitza Stark,[12,13] Tiong Yang Tan,[12,13] David R. Thorburn,[12,13] Susan M. White,[12,13] Maha S. Zaki,[18] Genomics Research to Elucidate the Genetics of Rare diseases (GREGoR) Consortium, Eric Vilain,[19] Monkol Lek,[3] Heidi L. Rehm,[1,4] and Anne O'Donnell-Luria[1,2,4,20,*]

Summary

Variants in the mitochondrial genome (mtDNA) cause a diverse collection of mitochondrial diseases and have extensive phenotypic overlap with Mendelian diseases encoded on the nuclear genome. The mtDNA is not always specifically evaluated in patients with suspected Mendelian disease, resulting in overlooked diagnostic variants. Here, we analyzed a cohort of 6,660 rare disease families (5,625 genetically undiagnosed [84%]) from the Genomics Research to Elucidate the Genetics of Rare diseases (GREGoR) Consortium, as well as other rare disease cohorts. Using dedicated pipelines to address the technical challenges posed by the mtDNA—circular genome, variant heteroplasmy, and nuclear misalignment—we called single nucleotide variants, small insertions/deletions, and large mtDNA deletions from exome and/or genome sequencing data, in addition to RNA sequencing data when available. Diagnostic mtDNA variants were identified in 10 previously genetically undiagnosed families (1 large deletion, 8 reported pathogenic variants, and 1 previously unreported likely pathogenic variant), as well as candidate diagnostic variants in a further 11 undiagnosed families. In one additional undiagnosed proband, detection of >900 heteroplasmic variants provided functional evidence of pathogenicity to a *de novo* variant in the nuclear gene *POLG* (DNA polymerase gamma), responsible for mtDNA replication and repair. Overall, mtDNA variant calling from data generated by exome and genome sequencing—primarily for nuclear variant analysis—resulted in a genetic diagnosis for 0.2% of undiagnosed families affected by a broad range of rare diseases, as well as the identification of additional promising candidates in 0.2%.

Introduction

Mitochondrial diseases (MDs) result from impaired cellular energy metabolism due to defects in the mitochondrial organelle.[1] Among rare genetic diseases, MDs are prime examples of the diagnostic challenge faced by geneticists, given vast genetic heterogeneity, with dual encoding on the nuclear and mitochondrial genome (mtDNA), and broad spectrum of associated clinical manifestations.[2]

The mtDNA is a circular, 16,569-base-pair, double-stranded DNA molecule present in hundreds to thousands of copies per cell. It encodes 13 protein-coding genes, 22 transfer RNA (tRNA) genes, and two ribosomal RNA (rRNA) genes, that are essential to mtDNA function. Pathogenic variants in the mtDNA are responsible for approximately 75% of adult-onset and 20%–25% of pediatric-onset MDs.[3] They span single nucleotide variants (SNVs), small insertions/deletions (indels), and large mtDNA deletions and are estimated to cause MDs in ≥1

[1]Program in Medical and Population Genetics, Broad Institute of MIT and Harvard, Cambridge, MA, USA; [2]Division of Genetics and Genomics, Boston Children's Hospital, Harvard Medical School, Boston, MA, USA; [3]Department of Genetics, Yale School of Medicine, New Haven, CT, USA; [4]Center for Genomic Medicine, Massachusetts General Hospital, Boston, MA, USA; [5]Hematology/Oncology, Bone Marrow Transplantation and Cellular Therapy, Pediatric Department, King Hussein Cancer Centre (KHCC), Amman, Jordan; [6]Department of Neurosciences, University of California, San Diego, San Diego, CA, USA; [7]Rady Children's Institute for Genomic Medicine, San Diego, CA, USA; [8]Children's National Research Institute, Washington, DC, USA; [9]Neuromuscular and Neurogenetic Disorders of Childhood Section, National Institute of Neurological Disorders and Stroke, National Institutes of Health, Bethesda, MD, USA; [10]Ocular Genomics Institute, Massachusetts Eye and Ear, Department of Ophthalmology, Harvard Medical School, Boston, MA, USA; [11]Department of Pathology, Boston Children's Hospital and Harvard Medical School, Boston, MA, USA; [12]Victorian Clinical Genetics Services, Murdoch Children's Research Institute, Flemington Road, Melbourne, Victoria, Australia; [13]Department of Paediatrics, University of Melbourne, Melbourne, Victoria, Australia; [14]Division of Hematology/Oncology, Boston Children's Hospital, Harvard Medical School, Boston, MA 02115, USA; [15]Department of Pediatric Oncology, Dana-Farber Cancer Institute, Harvard Medical School, Boston, MA 02215, USA; [16]Howard Hughes Medical Institute, Boston, MA 02115, USA; [17]Broad Institute of MIT and Harvard, Cambridge, MA 02142, USA; [18]Clinical Genetics Department, Human Genetics and Genome Research Institute, National Research Centre, Cairo 12311, Egypt; [19]Institute for Clinical and Translational Science, University of California, Irvine, Irvine, CA, USA
[20]Lead contact
*Correspondence: odonnell@broadinstitute.org
https://doi.org/10.1016/j.xhgg.2025.100441.
© 2025 The Authors. Published by Elsevier Inc. on behalf of American Society of Human Genetics.
This is an open access article under the CC BY license (http://creativecommons.org/licenses/by/4.0/).

per 5,000 individuals.[4,5] To date, 127 high-confidence confirmed pathogenic SNV/indel variants have been reported in the expert-curated database MITOMAP (https://www.mitomap.org/MITOMAP).[6] The majority of these variants cause disease in the heteroplasmic state, when the heteroplasmy level (HL) of mtDNA molecules carrying the variant exceeds a critical threshold in a susceptible tissue, typically reported as 60%–80%.[7] mtDNA heteroplasmy increases the complexity of genetic diagnosis, as the HL can vary from tissue to tissue. The HL is typically highest in post-mitotic tissues, such as skeletal muscle, heart, and brain, and is often lowest in rapidly replicating non-disease-affected tissues that are more readily accessible to sampling and routinely used first line for DNA testing, in particular blood and buccal cells.[8] In comparison, only a small number of homoplasmic variants have been associated with disease. These variants often demonstrate incomplete penetrance whereby only a subset of variant carriers manifest with the disease, as is commonly reported for the m.11778G>A (p.(Arg340His), NC_012920.1) variant causing Leber hereditary optic neuropathy (LHON) or lead to adult-onset and milder disease.[9]

Distinguishing mtDNA-encoded disease from other mitochondrial and non-mitochondrial nuclear-encoded diseases is clinically challenging due to the phenotypic heterogeneity of MDs and overlap with other nuclear-encoded neurological, neuromuscular, ophthalmological, and hematological diseases,[1,10] among others. It is, however, essential in determining the mode of inheritance to inform genetic counseling and provide accurate recurrence risk estimates and can be important in disease prevention, such as by egg donation, mitochondrial transfer, or preimplantation genetic diagnostics,[11] as well as to implement preventative measures and anticipatory care.

To reduce sequencing cost and streamline data analysis in rare disease diagnostics, the mtDNA may not always routinely undergo targeted sequencing and analysis unless an MD is clinically suspected. This potentially leads to cases of mtDNA-encoded MDs eluding detection when exome sequencing (ES) or genome sequencing (GS) are selected as the first-line diagnostic test.[12] Analysis of the mtDNA is possible in a holistic approach from ES[13,14] and GS data[15] by applying dedicated bioinformatic pipelines to call mtDNA SNVs/indels[16,17] and large mtDNA deletions.[18] For ES, probes can be added to the library preparation to capture the mtDNA at high coverage.[19] Alternatively, off-target reads can be analyzed, although this approach provides relatively low coverage of the mtDNA and is more likely to be enriched for nuclear DNA of mitochondrial origin (NUMTs).[14] In comparison, GS provides high coverage of the mtDNA due to the naturally high copy number of mtDNA molecules in cells.[15,16] mtDNA-specific bioinformatic pipelines navigate alignment issues created by the circular nature of the mtDNA, facilitate the detection of variants at a low HL (not possible in routine variant calling pipelines), and apply strategies to reduce the misalignment of NUMTs that can otherwise result in false-positive putative heteroplasmies. These pipelines have proven successful in the diagnosis of mtDNA-encoded disease in cohorts of suspected MDs and neurological diseases.[13–15,20]

Here, we apply mtDNA variant calling pipelines to GS, ES, and, where available, RNA sequencing data from a diverse collection of >6,500 rare disease families primarily sequenced through the Genomics Research to Elucidate the Genetics of Rare diseases (GREGoR) Consortium. We search for reported pathogenic variants and leverage recently released reference population databases of homoplasmic and heteroplasmic mtDNA variant allele frequencies (gnomAD v3[16] and HelixMTdb[21]) in combination with mtDNA-specific computational prediction tools and mitochondrial constraint metrics[22] for rare variant prioritization and assessment.

Subjects and methods

Sample selection

ES, GS, and RNA sequencing data (when available) from probands with a suspected rare disease and their affected and unaffected family members, recruited, sequenced, and phenotyped by the GREGoR Consortium (U07), were subject to mtDNA variant calling. In addition, samples from the Broad Institute Center for Mendelian Genomics (Broad CMG) that could not be part of GREGoR due to disease-specific consent, along with other rare disease cohorts sequenced in collaboration with the Broad CMG were included in the analysis. This resulted in a total of 14,282 samples from 7,282 families. ES libraries were generated using either Nextera capture (no mtDNA probes included) or Twist capture (with mtDNA probes included), and all samples were sequenced using Illumina instruments. Samples with a high level of contamination ($\geq$2% of haplogroup defining variants at 85%–99.8% HL) and/or a mean per sample mtDNA coverage of <20$\times$ were excluded from the study, resulting in 13,160 samples from 6,660 families for mtDNA variant analysis (Figure S1). This project was approved by the Mass General Brigham IRB (protocols #2016P001422 and #2013P001477).

mtDNA variant calling, haplogroup determination, and variant annotation

mtDNA variants were called from GS data using the mitochondria mode of GATK-Mutect2[16] and from ES/RNA sequencing using the MToolBox pipeline.[17] RNA was processed using a stranded polyA-tailed kit (Illumina). Large mtDNA deletions were called using MitoSAlt.[18] Variants were annotated with quality flags, functional consequence, reference population frequency, computational predictions, and mitochondrial constraint metrics.[22] Variants flagged as low quality were removed (see supplemental methods for more details).

Identifying pathogenic mtDNA variants

Variants with confirmed disease-causing status were extracted from MITOMAP ($n = 127$, last accessed October 2024).[6] Variants submitted as pathogenic/likely pathogenic (P/LP) with $\geq$2-star review status in association with primary MD were extracted from ClinVar ($n = 111$, last accessed October 2024).[23] This resulted in

a total of 152 unique reported P/LP variants for analysis (Table S1).

Identifying high-priority rare and potentially deleterious variants

mtDNA variants were filtered to (1) non-haplogroup defining variants (for the haplogroup of the respective sample), (2) non-synonymous variants, (3) rare variants detected in <1:50,000 individuals at homoplasmy in reference populations (gnomAD v3 and HelixMTdb) and with an allele count of ≤10 across all samples in the call set, and (4) variants meeting at least one of the following criteria for predicted deleteriousness: (1) predicted loss-of-function (frameshift, stop gained), (2) missense with an APOGEE2 score of >0.5[24] and/or HmtVar score ≥0.35[25]), (3) tRNA with a MitoTIP score of >12.66,[26] a PON-mt-tRNA probability score of ≥0.5,[27] and/or a HmtVar score of ≥0.35, and (4) within an area of regional constraint or at a nucleotide position with high mitochondrial local constraint (MLC score of ≥0.75)[22] (see supplemental methods for more details).

Variant interpretation and confirmation

Identified variants were clinically evaluated as either (1) diagnostic—a variant classified as P/LP according to the ClinGen Variant Curation Expert Panel mtDNA specifications of the American College of Medical Genetics and Genomics and Association of Molecular Pathologists (ACMG/AMP) standards and guidelines for variant interpretation,[28] that explains the proband's phenotype, is detected at a clinically relevant HL, for which the multidisciplinary analysis team and referring clinician consider the variant causative, and clinically confirmed in a CLIA certified laboratory, or (2) candidate—a reported P/LP variant or high priority variant of uncertain significance (VUS) that may explain the proband's phenotype but requires additional evidence to establish causality and/or pathogenicity, or (3) a pathogenic variant of undetermined clinical relevance—a reported P/LP variant that does not explain the individual's phenotype and/or is known to demonstrate incomplete penetrance at near homoplasmy.

Phenotype data analyses

Phenotype data were collected as human phenotype ontology (HPO) terms. Each reported HPO term was mapped in the ontology to "phenotypic abnormality" (HP: 0000118) and annotated with all intermediate terms. The objective clinical likelihood of the proband having a disease of mitochondrial etiology was calculated by the Mitochondrial Disease Criteria (MDC) score,[29] adapted for use with HPO terms.[10] MDC scores were stratified into unlikely (score 0–1), possible (score 2–4), probable (score 5–7), and definite (score 8–12) MD.

Results

Cohort description

In total, 6,660 families were included in our analysis after sample-level quality filtering (see methods). The majority (5,625 [84%]) were genetically undiagnosed following nuclear analysis of ES/GS (Figure 1A). Among the solved families were three that had already had an mtDNA-encoded diagnosis returned by targeted mtDNA sequencing, used as positive controls for our variant calling and analysis pipelines. Data from multiple sequencing methods (ES, GS, and/or RNA sequencing) were analyzed for 164 probands (Figure 1B).

Most samples were derived from DNA extracted from blood from probands with pediatric onset of disease, and therefore less likely to carry mtDNA variants restricted to post-mitotic tissues. A median of three non-redundant HPO terms were reported per proband (range, 0–121). For 5,192 probands (78%), ≥1 reported HPO term overlapped with a term associated with MDs, according to the MDC score,[29] spanning muscular, neurological, multisystem, metabolic, imaging, and histopathology terms (Figure 1C). Based on the combination of these phenotypes and applying the MDC, 1.2% of probands had a probable or definite likelihood of an MD, 35% possible, and 64% unlikely, prior to genetic analysis. These figures indicate a low prior probability of an MD based on the clinical phenotype for most families in our study.

mtDNA coverage and variant detection summary

The mean per-base mtDNA coverage for GS and RNA sequencing was high (GS mean, 4,416×; RNA sequencing mean, 5,894×). The coverage by ES depended on capture selection. ES (Twist) provided high coverage (mean, 6,315×) by adding mtDNA probes, whereas ES (Nextera) provided low coverage from off-target reads (mean, 47×) (Figure 2).

A mean of 40 mtDNA variants were called at ≥1% HL per sample, of which 26 per sample passed our quality filters (see supplemental methods). Collectively, 6,960 unique variants were detected, spanning 6,069 of the 16,569 nucleotide positions of the mtDNA (37%). Most were detected at near homoplasmy (≥95% HL) and were known haplogroup defining variants (mean, 23 per sample), which are unlikely to be causal of MDs. A summary of the counts of high-quality variants per proband sample for analysis by data type is displayed in Table S2.

Maternal samples were available for 3,056 probands (46%), allowing comparison of HL between generations that can be informative for clinical interpretation. Overall, 99% (80,753/81,772) of variants in the probands were detected in the maternal sample of the corresponding data type, >99% of homoplasmic variants (77,449/77,767), and 82% of heteroplasmic variants (3,304/4,005). A small number of these variants demonstrated either a potentially clinically relevant positive heteroplasmic shift, from below to above the typical disease-causing threshold of 60% (147/80,753 [0.18%]) or negative heteroplasmic shift, from >60% to <60% HL (85/80,753 [0.11%]), although the majority were more neutral (Figure S2). Variants detected in the proband only (1,019/81,772 variants [1.2%]) may be *de novo*, somatic, or present at undetectable levels in the maternal tissue sampled. More than one-third of the variants detected in the proband only were at a HL of ≥60% (388/1,019 [38%]) and, when predicted to be deleterious, are promising candidates for sporadic disease in the proband.

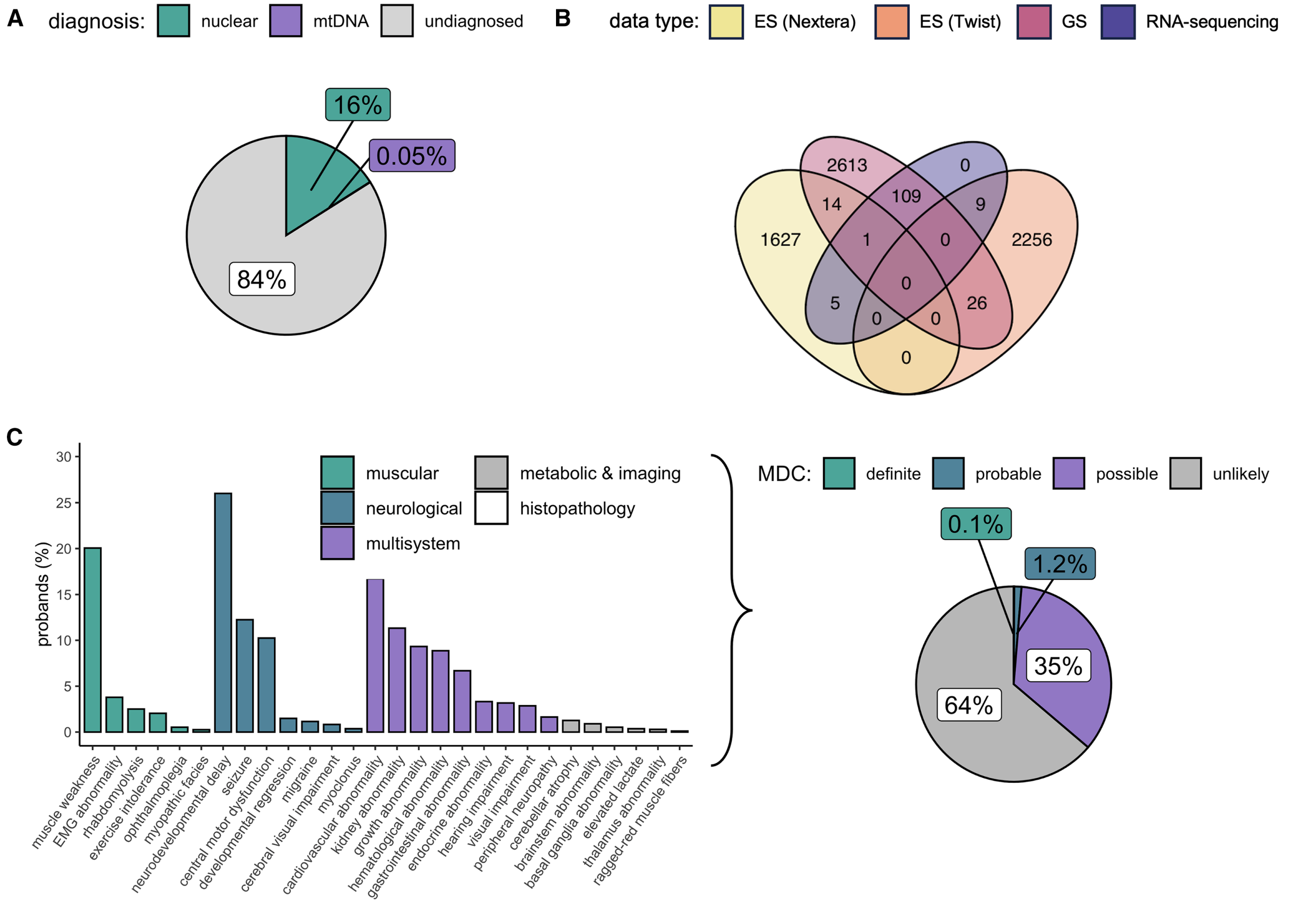

Figure 1. Study cohort overview

(A) Diagnostic status of probands following nuclear variant analysis by ES and/or GS, prior to the analysis of mtDNA variants in this study. For three probands (0.05%), diagnostic variants in the mtDNA had already been detected by targeted mtDNA sequencing.
(B) Proband samples by sequencing type, demonstrating overlap in data type for analysis.
(C) Frequency of HPO terms indicative of MD in the probands (displaying terms reported in ≥5 probands) and the resultant MDC classification of clinical likelihood of an MD.[29]

Detection of reported P/LP variants

Reported P/LP variants were detected in a total of 59 probands. Large mtDNA deletions were detected in two of these probands (Figure 3A) and pathogenic mtDNA SNVs or small indels, reported in MITOMAP as confirmed disease causing and/or reported in ClinVar as P/LP with ≥2-star review status, were identified in the remaining 57 probands (24 different variants) at ≥5% HL (Figure 3B). In total, nine new diagnoses were made (including one large deletion), two plausible candidate diagnoses were identified, and all three of the known mtDNA diagnoses in the cohort (including one large deletion) were re-identified (Table 1). In the remaining 45 probands, the pathogenic variants were of undetermined clinical relevance.

Non-diagnostic pathogenic variants may be detected in individuals, including those in reference populations, at a HL below the disease-causing threshold (typically reported at ≥60%, although dependent on the specific variant and tissue) and at near homoplasmy when the variant demonstrates incomplete penetrance or is associated with adult-onset or mild disease.[13,31] Stratifying the detected pathogenic variants by reported incomplete penetrance at near homoplasmy, we find all P/LP variants at a high HL, without reports of incomplete penetrance to be diagnostic, with the proband's phenotype being in keeping with reported phenotypes for the variant (Figure 3B). In contrast, we detected many non-diagnostic P/LP variants of undetermined clinical relevance at high HL that are reported to be incompletely penetrant.

The most frequently detected non-diagnostic pathogenic variants were (1) m.1555A>G (NC_012920.1) in *MT-RNR1* (10 probands) associated with susceptibility to aminoglycoside ototoxicity, (2) m.3243A>G (NC_012920.1) in *MT-TL1* (10 probands) associated with mitochondrial encephalopathy, lactic acidosis, and stroke-like episodes (MELAS), although highly phenotypically heterogeneous, and (3) m.11778G>A (p.(Arg340His), NC_012920.1) in *MT-ND4* (7 probands) associated with LHON. Both m.1555A>G and m.11778G>A are known to demonstrate incomplete penetrance at near homoplasmy.[13] There are also numerous reports of asymptomatic individuals with m.3243A>G at a high HL in blood (≥60%).[32]

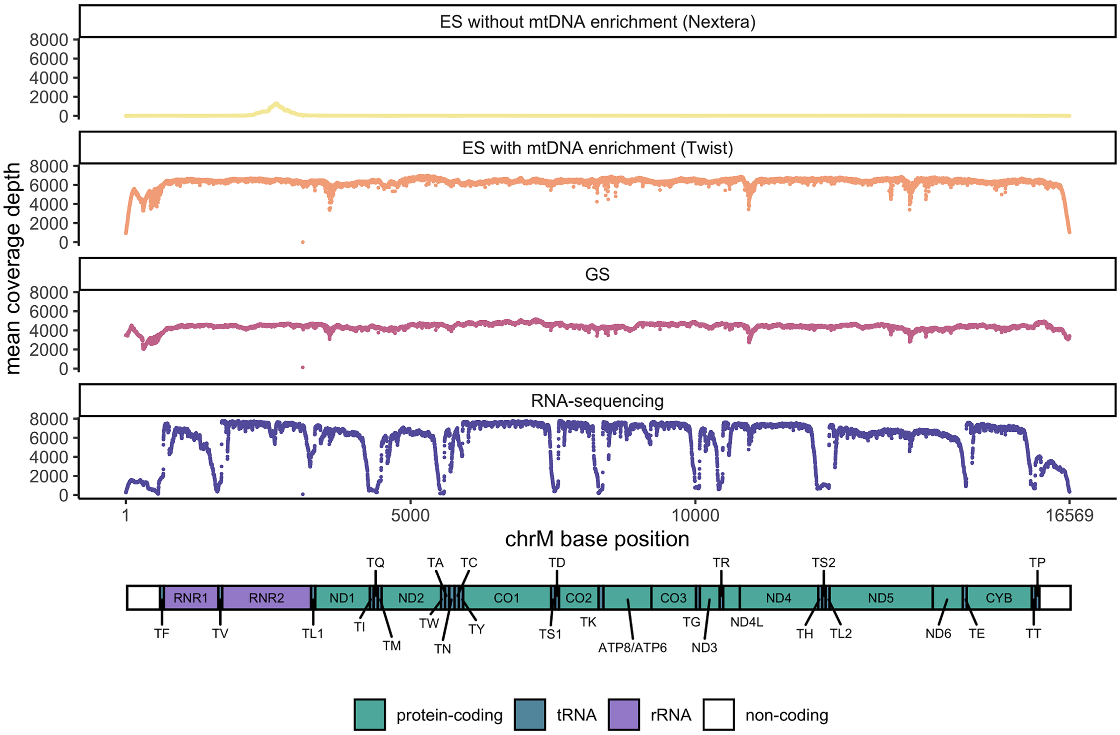

Figure 2. mtDNA coverage by data type
Mean per-base mtDNA coverage by chrM base position. Schematic of a linearized mtDNA, demonstrating lowest coverage over the artificial break in the mitochondrial D loop and low RNA sequencing coverage of the tRNA genes due to lack of a stable 3′-poly(A) tail for enrichment.[30]

Detection of rare potentially deleterious variants

We next sought to investigate whether other rare mtDNA variants may be causing disease. To prioritize variants with high disease-causing potential, we applied stringent filtering by function, frequency in reference populations, predicted deleteriousness, and mitochondrial constraint metrics (see methods). In total, 555 variants were prioritized in 518 probands (0.08 per proband across all analyzed probands) (Figures 4A and 4B). Each variant was carefully reviewed for potential relevance to phenotype and, if considered of diagnostic interest, was classified according to the ACMG/AMP standards and guidelines for mitochondrial DNA variant interpretation. This clinical evaluation led to one new diagnosis and one candidate diagnosis classified as LP by the mtDNA-specifications of the ACMG/AMP,[28] in addition to eight high-priority candidates classified as VUS (Table 2).

The new LP diagnosis is a *de novo* m.15347C>T (p.(His201Tyr), NC_012920.1) variant in *MT-CYB*, detected at 19% HL in the blood of a genetically undiagnosed male proband (P15) who presented in the neonatal period with progressive hypertrophic cardiomyopathy, renal cortical dysplasia, hyperinsulinemic hypoglycemia, and elevated lactate in both the serum and cerebrospinal fluid. His phenotype was progressive with death at 6 months of age. *MT-CYB* encodes a subunit of mitochondrial complex III. The p.(His201) amino acid position has high conservation (MITOMASTER 100% across species) and is in an area of regional missense constraint,[22] with this residue thought to be critical for ubiquinone binding.[33] The variant has consistently deleterious computational predictions (APOGEE2 0.64, HmtVar 0.84), is absent in reference populations at both homo- and heteroplasmy (gnomAD v3 and HelixMTdb), and has not previously been reported in clinical cases. Follow-up by targeted whole mtDNA analysis on DNA extracted from heart tissue found the variant to be present at 87.5% HL. The variant was initially classified as a VUS. Subsequent segregation testing of maternal DNA extracted from both blood and urine was negative, suggesting the variant to be *de novo*. Functional studies were also performed, demonstrating the variant to have a deleterious effect on levels of the MT-CYB protein and on complex III activity and protein levels in affected tissues (heart and muscle, data not shown). With these additional lines of evidence for pathogenicity, the variant was reclassified as LP (PS2, PS3_Supporting, PM2_Supporting, PP3_Supporting) and was returned to the family to inform family planning.

The LP candidate diagnosis is a *de novo* m.16023G>A (NC_012920.1) variant in *MT-TP*, detected at 15% HL in blood in a male proband (P16) presenting in infancy with unexplained seizures, failure to thrive, pancreatic exocrine insufficiency, neutropenia, anemia, lethargy, and recurrent infections typically with hemodynamic instability requiring intensive care admission. The variant was absent in the mother's GS data. *MT-TP* encodes a

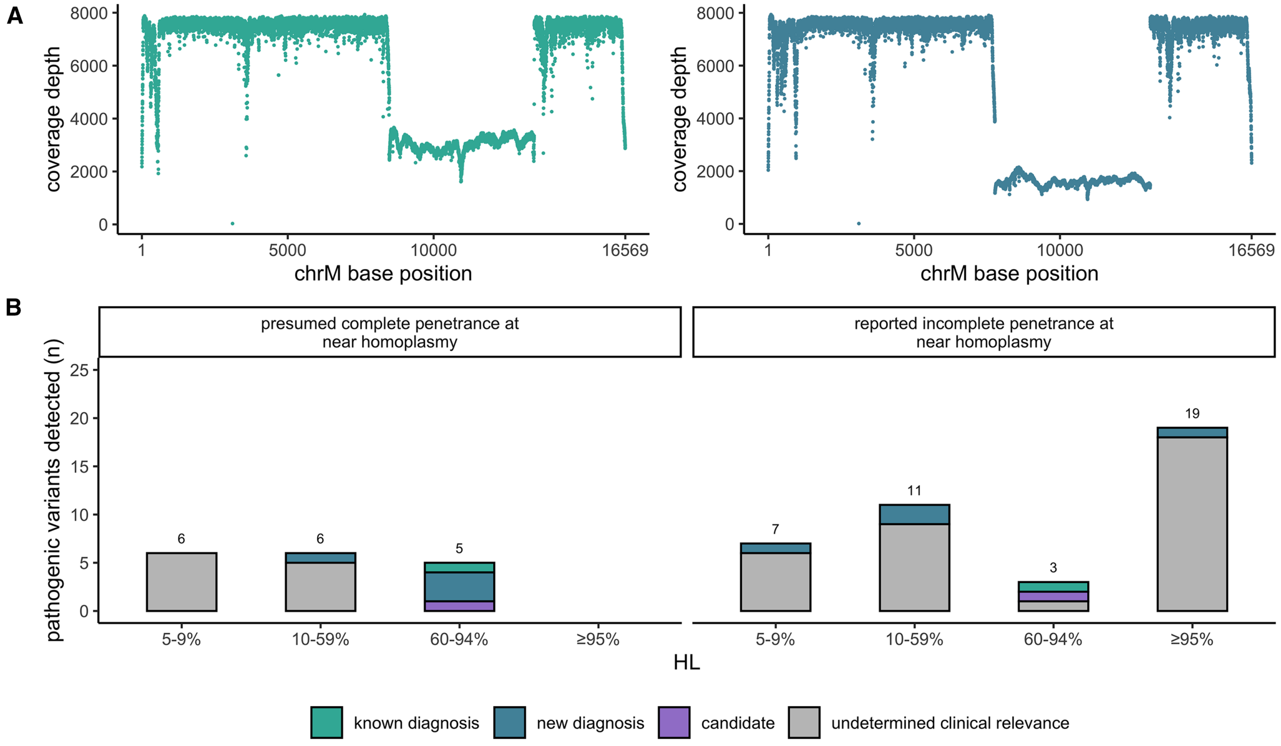

Figure 3. Detection of pathogenic mtDNA variants
(A) Coverage by chrM base position in two samples with large mtDNA deletions (green [known diagnosis] and blue [new diagnosis]).
(B) Pathogenic variants detected across all probands stratified by penetrance at near homoplasmy.[13]

mitochondrial tRNA. The variant has consistently deleterious computational predictions (MitoTIP 17.6, HmtVar 0.65, and PON-mt-tRNA 0.8). It is absent in reference populations at homoplasmy and is rare at heteroplasmy (absent in gnomAD v3 and a heteroplasmic allele count of three in HelixMTdb with a maximum detected HL of 15%). The variant is listed in MITOMAP with reported status, and was previously reported in two unrelated probands. In the first proband with migraine, pigmentary retinopathy, deafness, leukariosis on magnetic resonance imaging (MRI), cytochrome c oxidase-negative fibers and ragged red fibers on muscle biopsy (proband HL 9% in blood, 86% in muscle, and 36% in urine; mother HL 1% in blood and 7% in urine) the variant is reported to be diagnostic. In the second proband with liver dysfunction, urticaria, developmental delay, and fatigue (HL 2% in muscle), the variant remains of undetermined clinical relevance.[34,35] The variant has been functionally validated by gold standard single-fiber analysis.[34] Given these lines of evidence, the variant is classified as LP (PM6, PS3_Supporting, PS4_Supporting, PM2_Supporting, PP3_Supporting). At present, we consider this LP variant as a strong candidate diagnosis for the proband, rather than diagnostic, given the low HL in blood (15%) and unavailability of additional tissues for testing at this time. Clinical follow-up by sequencing of additional tissues from both the proband and the mother are needed to determine if this variant is diagnostic for the family. The remaining eight high-priority candidates are reported in Table 2.

For genetically undiagnosed probands with a probable-definite MDC score (65 probands), we additionally reviewed all rare, non-synonymous, non-haplogroup-defining mtDNA variants, regardless of computational prediction and constraint metrics; however, this did not yield any further high-priority candidates.

Detection of excess heteroplasmic variants provides functional evidence of pathogenicity for a *de novo* variant in the proofreading exonuclease domain of *POLG*

As a part of our analysis, we counted the number of heteroplasmic variant calls per proband at ≥1% HL. This analysis revealed an outlier proband (P25) in the ES (Twist) dataset with 941 heteroplasmic variants (910 SNVs, 31 indels), compared with a mean of 10.1 per sample (Figure 5A). Only 12 of the 941 variants were detected in the corresponding maternal sample, indicating either an issue with sample quality (despite passing our sample-level coverage and contamination filters) or pointing toward an underlying defect in the replication and repair of the mtDNA leading to high rates of somatic SNVs. Analysis of ES from the proband and both unaffected parents for causal nuclear variants had detected a heterozygous, previously unreported, *de novo* missense variant that was initially of uncertain significance in the nuclear gene

Table 1. Diagnostic and candidate reported pathogenic mtDNA variants

Proband ID	Sample data type	Gene symbol	Variant (Consequence)	MITOMAP status	ClinVar status	Sample HL (alt/ref reads)	Inheritance	Reported phenotype	Proband's phenotype	Proband's MDC (score)	Diagnostic outcome
P1	ES	*MT-ND1*	m.3946G>A, p.(Glu214Lys) (missense)	rptd	P/LP	70% (2,924/1,247)	–	MELAS	infantile spasms, cortical visual impairment, and severe NDD	possible (3)	known diagnosis
	RNA-seq					68% (4,720/2,231)					
P2	ES	*MT-ATP6*	m.8993T>G, p.(Leu156Arg) (missense)	cfrm	P	87% (5,473/847)	–	NARP, LSS, MILS, LHON	retinitis pigmentosa	unlikely (1)	known diagnosis
P3	ES	-	m.8470_13446del4977 (single deletion)	–	–	70% (4,701/1,958)	–	Pearson syndrome	Pearson syndrome and DBA	possible (3)	known diagnosis
P4	ES	-	m.7773_13094del5322 (single deletion)	–	–	82% (5904/1316)	–	Pearson syndrome	Pearson syndrome and CSA	possible (4)	new diagnosis
P5	ES	*MT-TL1*	m.3243A>G (tRNA)	cfrm	P/LP	8% (490/5,634)	–	MELAS, LSS, MIDD, SNHL, CPEO, myopathy, FSGS, and ASD	retinitis pigmentosa	unlikely (1)	new diagnosis
P6	GS	*MT-TL1*	m.3243A>G (tRNA)	cfrm	P/LP	10% (501/5,014)	–	MELAS, LSS, MIDD, SNHL, CPEO, myopathy, FSGS, and ASD	fatigue, periodic fevers, headache, recurrent fever, tinnitus, recurrent episodes of infection, intermittent rashes, significant nausea	possible (2)	new diagnosis
P7	ES	*MT-TL1*	m.3243A>G (tRNA)	cfrm	P/LP	11% (484/3,959)	–	MELAS, LSS, MIDD, SNHL, CPEO, myopathy, FSGS, and ASD	retinitis pigmentosa	unlikely (1)	new diagnosis
P8	ES	*MT-TL1*	m.3243A>T (tRNA)	cfrm	P/LP	33% (1,384/4,194)	transmitted (1.4% HL in maternal sample)	myopathy, MELAS, SNHL, CPEO	mitochondrial myopathy (RRF, COX− fibers), fatigue, growth delay	probable (7)	new diagnosis
P9	GS	*MT-TA*	m.5591G>A (tRNA)	rptd	P	86% (4,253/710)	–	myopathy	mitochondrial myopathy (RRF, COX− fibers), exercise intolerance, elevated CK, headaches, ID, tachycardia	definite (8)	new diagnosis
P10	GS	*MT-ATP6*	m.8969G>A, p.(Ser148Asn) (missense)	cfrm	LP	70% (3,646/1,540)	–	MLASA, IgG nephropathy	LSS, hypotonia, FTT, episodic vomiting	probable (6)	new diagnosis
P11	ES	*MT-ATP6*	m.8993T>C, p.(Leu156Pro) (missense)	cfrm	P	99% (7,018/71)	transmitted (90% HL in maternal sample)	NARP, LSS, MILS, LHON	hypotonia, cerebellar atrophy, speech delay	possible (3)	new diagnosis

(*Continued on next page*)

Table 1. Continued

Proband ID	Sample data type	Gene symbol	Variant (Consequence)	MITOMAP status	ClinVar status	Sample HL (alt/ref reads)	Inheritance	Reported phenotype	Proband's phenotype	Proband's MDC (score)	Diagnostic outcome
P12	GS	*MT-ATP6*	m.9134A>G, p.(Glu203Gln) (missense)	rptd	LP	82% (4,921/6,038)	*de novo*	IUGR, hypotonia, HCM, and LA	SGA, LA, NDD, infantile spasms	probable (5)	new diagnosis
P13	GS	*MT-TF*	m.591C>T (tRNA)	rptd	P/LP	72% (4,388/963)	transmitted (36% HL in maternal sample)	renal tubulopathy	renal tubulopathy, ID, NDD, short stature	possible (4)	candidate
P14	GS	*MT-TL1*	m.3243A>T (tRNA)	cfrm	P/LP	70% (3,373/4,819)	transmitted (9% HL in maternal sample)	myopathy, MELAS, SNHL, CPEO	SNHL, cerebral palsy, holoprosencephaly[a]	possible (2)	candidate

ASD, autism spectrum disorder; cfrm, confirmed; CLIA, Clinical Laboratory Improvement Amendments; CMS, congenital myasthenic syndrome; COX, cytochrome c oxidase; CPEO, chronic progressive external ophthalmoplegia; CSF, cerebrospinal fluid; DBA, Diamond Blackfan anemia; EM, encephalomyopathy; FSGS, focal segmental glomerulosclerosis; FTT, failure to thrive; HCM, hypertrophic cardiomyopathy; IUGR, intrauterine growth restriction; LA, lactic acidosis; LSS, Leigh syndrome spectrum; MELAS, mitochondrial encephalopathy, lactic acidosis, and stroke-like episodes; MIDD, maternally inherited diabetes and deafness; MLASA, mitochondrial myopathy, lactic acidosis and sideroblastic anemia; NARP, neuropathy, ataxia, retinitis pigmentosa; NDD, neurodevelopmental delay; OXPHOS, oxidative phosphorylation; rptd, reported; SNHL, sensorineural hearing loss. RefSeq accession number and version number of mtDNA sequence: NC_012920.1.

[a]LP variant in *ZIC2* explains the proband's holoprosencephaly phenotype, *MT-TL1* is a candidate for the SNHL and would be a dual diagnosis.

POLG, encoding DNA polymerase gamma (NM_002693.3: c.592G>A, p.(Asp198Asn)), responsible for mtDNA replication and repair. This variant affects the p.(Asp198) residue, an essential catalytic residue involved in the POLG protein's proofreading exonuclease activity, and is predicted to be deleterious (REVEL score of 0.94) (Figure 5B). The variant is absent in the gnomAD v4 reference population. In reported cellular models, mutagenesis of p.(Asp198) to p.(Asp198Ala) abolishes the exonuclease activity of POLG[36] and, in keeping with the finding of a high heteroplasmic variant detection rate in our proband, results in the accumulation of somatic SNVs in the mtDNA.[37] Similarly, the *POLG* mutator mouse that is lacking the mtDNA proofreading exonuclease activity rapidly accumulates somatic mtDNA SNVs.[38] The proband's p.(Asp198Asn) *POLG* variant is absent in reference population databases. He is currently 12 years of age with congenital sideroblastic anemia (CSA) (diagnosed at 7 years of age), leukopenia, moderate neutropenia, and lymphopenia, with no associated history of recurrent infections. He has short stature (first centile) as well as cognitive and learning disabilities, and his brain MRI demonstrates polymicrogyria. He was recently diagnosed with early type 1 diabetes mellitus. A CSA phenotype is currently reported once in association with a heterozygous *POLG* variant in the literature,[39] yet is highly consistent with both nuclear- and mtDNA-encoded MD.[40] Moreover, among the many heteroplasmic mtDNA variants detected in the proband was a rare, somatic, predicted deleterious (APOGEE2 0.76) missense variant in *MT-ND1* (m.3976T>C, p.(Phe224Leu), NC_012920.1). The p.(Phe224) amino acid position has high conservation (MITOMASTER 97.8% across species) and is in an area of regional missense constraint, and the nucleotide position (m.3976T) has high mitochondrial local constraint (MLC score of 0.86). *MT-ND1* encodes a subunit of mitochondrial complex I. The variant is detected at 21% HL and may explain the hematological manifestation of disease in this proband (Figure 5C). For the two GS samples with excess heteroplasmic variants, no potentially causal rare variants were identified in genes involved in mtDNA replication and repair and maternal data were not available to determine if the variants are inherited or *de novo*. All three samples with excess heteroplasmic variants passed contamination quality checks (see supplemental methods).

Diagnostic detection rate

In summary, across the 6,660 probands included in our analysis, a total of 614 variants were prioritized for clinical evaluation, spanning reported pathogenic SNV and indel variants (57 total), large mtDNA deletions (2 total), and prioritized rare potentially deleterious variants (555 total), or approximately 1 variant per 10 probands. Our variant calling and analysis pipelines re-identified all three known mtDNA diagnoses in the previously solved families with targeted mtDNA sequencing (including one large mtDNA

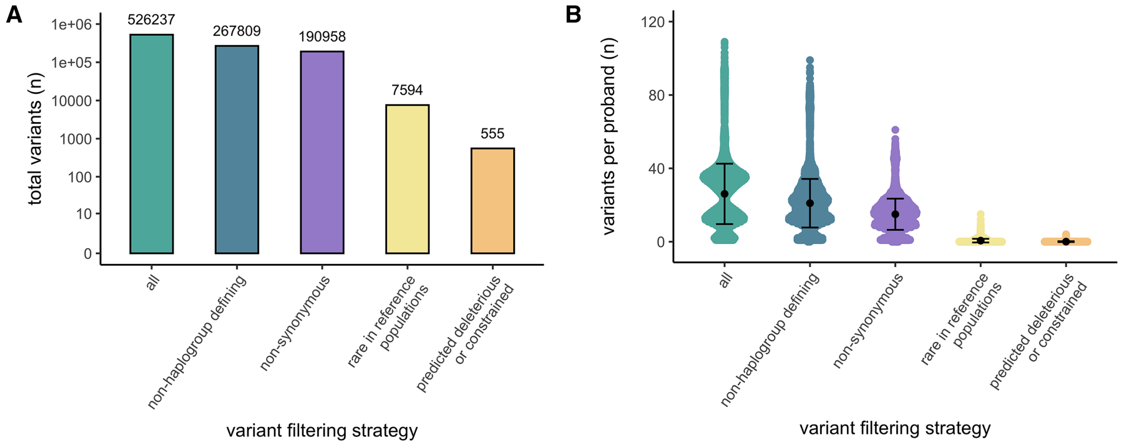

Figure 4. Prioritization of rare potentially deleterious variants for clinical evaluation
(A) Number of variants in total and (B) per proband for clinical evaluation, displayed with the mean and standard deviation.

deletion) and established 10 additional diagnoses among the undiagnosed families. Candidate diagnoses were also identified in 11 probands from undiagnosed families that remain under investigation (e.g., sequencing of additional tissues and sequencing additional maternal family members). Together with the *POLG* diagnosis, findings from the analysis of the mtDNA resulted in a diagnostic uplift of 0.2% (11/5,625) in undiagnosed families with a generally low prior probability of an MD, as well as the identification of additional promising candidates.

Discussion

We evaluated the diagnostic yield of calling mtDNA variants from ES and GS data that had already been generated and analyzed for suspected Mendelian (nuclear) causes of disease. This followed the rationale that mtDNA-encoded MDs may be overlooked in the differential diagnoses when referring a family for genetic testing, due to extensive overlap with seemingly Mendelian phenotypes, including nuclear causes of MDs that are more frequent in children.

We provided a diagnosis to a total of 11 previously undiagnosed families, in addition to identifying candidates in a further 11, presenting clinically with a broad range of rare diseases. Many of our candidates were identified in probands with CSA, a phenotype with a very high probability of being an MD, either associated with nuclear-encoded mitochondrial proteins or encoded by the mtDNA.[40] According to our calculation of the MDC based on available HPO terms, many candidates were also detected in probands with phenotypes indicating an unlikely or possible likelihood of MD including retinal disease, a phenotype for which nuclear variants are more frequently investigated.[40,41] Our diagnostic findings demonstrate the value of adding mtDNA analysis to routine ES/GS data analysis for probands where a nuclear-encoded mitochondrial cause is considered likely, as well as for those with a low prior probability of MD. Moreover, for all probands with maternal data available, the diagnostic or candidate variants were found to either arise *de novo*, as reported for ∼20% of mtDNA-encoded disease,[42] or to have been transmitted from the unaffected mother with an increase in HL, presumably at the point of the mitochondrial bottleneck in development.[38] Therefore, the absence of a maternal family history should not exclude the suspicion of an mtDNA-encoded disease. The diagnostic uplift of 0.2% among undiagnosed families was in line with expectation based on published studies demonstrating diagnostic rates of 0.1% (11/11,424)[14] in individuals enriched for neurological diseases and 1.6% (5/319)[15] to 1.8% (38/2,111)[13] in individuals with a higher clinical suspicion of MD.

We also report on the overall rate of pathogenic mtDNA variant detection among sequenced probands by HL threshold, finding P/LP variants in many probands to be secondary findings for conditions or genes not included on the ACMG recommended reporting list. The detection of high HL pathogenic variants of undetermined clinical relevance was mostly accounted for by incomplete penetrance, such as variants conveying a risk of LHON.[9] This adds to our understanding of how frequently secondary findings can be expected when routinely analyzing the mtDNA from ES/GS data. We also detected many low HL pathogenic variants of undetermined clinical relevance. Due to inconsistency of the respective proband's phenotype with reported phenotypes, we did not pursue these variants further (e.g., by sequencing additional tissues).

Our study provides a glimpse into the added value of searching for other rare, potentially deleterious mtDNA variants in a diverse rare disease cohort that has not been assessed in earlier studies. By stringent filtering for high-quality variant calls at a low population frequency, followed by prioritization for predicted deleterious consequence and areas of mitochondrial constraint, we identified one new diagnosis and nine promising candidates in undiagnosed families with phenotypes within the MD

Table 2. Diagnostic and candidate rare potentially deleterious mtDNA variants

Proband ID	Sample data type	Gene symbol	Variant (Consequence)	MITOMAP status	ClinVar status	Sample HL (alt/ref reads)	Heteroplasmic AF (AC, max observed HL)	Computational evidence of deleteriousness	Mitochondrial constraint	Transmission	Reported phenotype	Proband's phenotype	Proband's MDC (score)	ACMG classification	Diagnostic outcome
P15	ES	*MT-CYB*	m.15347C>T, p.(His201Tyr) (missense)	–	–	19% (7/30)	gnomAD: absent Helix: absent	APOGEE 0.64, HmtVar 0.84	regional missense constraint MLC:0.53	*de novo*	–	HMC, LA, elevated CSF lactate	probable (6)	LP	new diagnosis
P16	GS	*MT-TP*	m.16023G>A (tRNA)	rptd	VUS	15% (1,584/ 8,977)	gnomAD: absent Helix: 1.53e−05 (3, 15%)	MitoTIP 17.6, HmtVar 0.65, PON-mt-tRNA 0.8	MLC:0.73	*de novo*	migraine, pigmentary retinopathy, deafness, leukoaraiosis	seizures, FTT, neutropenia, anemia	possible (3)	LP	candidate
P17	GS	*MT-CO1*	m.6853G>C, p.(Gly317Ala) (missense)	–	–	6% (308/ 4,829)	gnomAD: absent Helix: absent	APOGEE 0.52, HmtVar 0.8	MLC:0.87	transmitted (2% HL in maternal sample)	–	seizures, speech and language delay, myopathy	possible (4)	VUS	candidate
P18	GS	*MT-TK*	m.8328G>A (tRNA)	rpt	VUS	6% (537/ 8,952)	gnomAD: absent Helix: 5.10e−06 (1, 14%)	MitoTIP 117.5, PON-mt-tRNA 0.92	MLC:0.62	*de novo*	encephalopathy/ EXIT with myopathy and ptosis	hypotonia, stridor, feeding difficulties, laryngomalacia, chronic constipation, fatigue, disturbed sleep, aerophagia, reflux, asthma, periodic limb movement, muscle fatigue, loss of skill, plagiocephaly, neck flexion weakness	possible (3)	VUS	candidate
P19	GS	*MT-ATP8*	m.8424T>C, p.(Leu20Pro) (missense)	rptd	–	96% (2,265/ 2,359)	gnomAD: absent Helix: 5.10e−06 (1, 15%)	APOGEE2 0.55, HmtVar 0.85	MLC:0.01	*de novo*	–	LSS, FTT, hypotonia, seizures, regression	probable (5)	VUS	candidate
	RNA-seq					98% (7,560/ 195)									
P20	ES	*MT-ATP8*	m.8570T>C, p.(Ter669Gln) (stop-loss)	–	–	95% (6,8687, 229)	gnomAD: 3.54e−05 (2, 28%) Helix: absent	LoF	MLC:0.03	–	–	CSA	possible (4)	–	candidate

(*Continued on next page*)

Table 2. *Continued*

Proband ID	Sample data type	Gene symbol	Variant (Consequence)	MITOMAP status	ClinVar status	Sample HL (alt/ref reads)	Heteroplasmic AF (AC, max observed HL)	Computational evidence of deleteriousness	Mitochondrial constraint	Transmission	Reported phenotype	Proband's phenotype	Proband's MDC (score)	ACMG classification	Diagnostic outcome
P21	ES	*MT-ATP6*	m.8611C>A, p.(Leu29Met) (missense)	–	–	100% (5,651/0)	gnomAD: absent Helix: absent	APOGEE 0.45, HmtVar 0.71	MLC:0.01	–	–	NDD, cerebellar atrophy, strabismus	possible (3)	VUS	candidate
P22	ES	*MT-ATP6*	m.8797T>C, p.(Ser91Pro) (missense)			50% (3,694/ 7,387)	gnomAD: absent Helix: absent	APOGEE 0.39 HmtVar 0.85	MLC:0.15	–	–	LSS	possible (3)		candidate
P23	ES	*MT-TH*	m.12197C>T (tRNA)	–	–	100% (7,776)	gnomAD: 1.77e−05 (1, 17%) Helix: 1.02e−05 (2, 29%)	MitoTIP 14.6, PON-mt-tRNA 0.62	MLC:0.27	transmitted (57% HL in maternal sample)	–	CSA, SNHL, ID, motor delay	possible (4)	VUS	candidate
P24	ES	*MT-TH*	m.12198T>C (tRNA)	–	–	87% (6,707/ 1,002)	gnomAD: absent Helix: 5.10e−06 (1, 9%)	MitoTIP 17.9, PON-mt-tRNA 0.53	MLC:0.28	–	–	CSA	possible (4)	VUS	candidate

ASD, autism spectrum disorder; CMS, congenital myasthenic syndrome; CPEO, chronic progressive external ophthalmoplegia; CSF, cerebrospinal fluid; DBA, Diamond Blackfan anemia; EM, encephalomyopathy; FSGS, focal segmental glomerulosclerosis; FTT, failure to thrive; HCM, hypertrophic cardiomyopathy; LA, lactic acidosis; LSS, Leigh syndrome spectrum; MELAS, mitochondrial encephalopathy, lactic acidosis, and stroke-like episodes; MIDD, maternally inherited diabetes and deafness; MLASA, mitochondrial myopathy, lactic acidosis and sideroblastic anemia; NARP, neuropathy, ataxia, retinitis pigmentosa; NDD, neurodevelopmental delay; RP, retinitis pigmentosa; SNHL, sensorineural hearing loss.
RefSeq accession number and version number of mtDNA sequence: NC_012920.1.

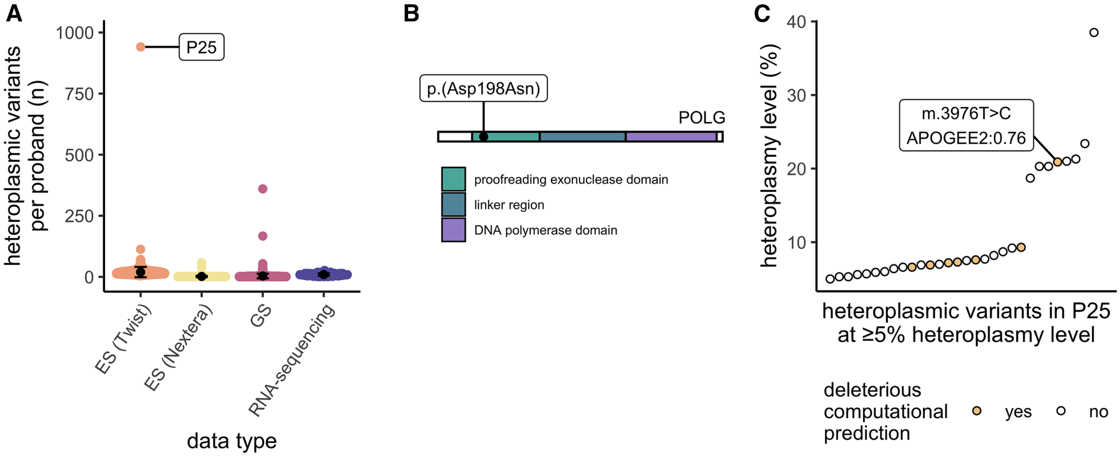

Figure 5. ***De novo*** **variant in** ***POLG*** **results in high numbers of somatic heteroplasmic mtDNA variants**
(A) Number of high-quality mtDNA variants per proband by data type, demonstrating P25 as an outlier sample with >900 variants.
(B) Schematic representation of POLG. The *de novo POLG* variant in P25 is located within the proofreading exonuclease domain of the protein.
(C) Heteroplasmic mtDNA variants detected in P25 at ≥5% HL indicating those with deleterious computational prediction.

spectrum. The majority of the candidate variants formally remain as VUS according to the mtDNA specifications of the ACMG/AMP guidelines for variant interpretation,[28] and additional evidence of pathogenicity is required to reach P/LP status, such as sequencing additional tissues, pursuing segregation studies in maternal relatives, further investigating blood and/or cerebrospinal fluid metabolite profiles, brain imaging, tissue histology, and respiratory chain enzymology, as well as functional studies. Candidates have been returned to local clinical or research teams in case additional study is warranted and possible, but it is beyond the scope of this study.

The field standard for assessing the population frequency of mtDNA variants is to use the homoplasmic allele frequency,[28] provided by long-standing databases such as MITOMAP, HmtDB, and MSeqDR.[6,25,43] The majority of pathogenic mtDNA variants are, however, heteroplasmic in nature, and, although homoplasmic frequencies have been highly valuable in providing evidence for or against heteroplasmic variants that are also seen at homoplasmy, they have not provided a complete picture as they have not captured the heteroplasmic frequency.[2] In our analyses, we leveraged recently released reference population databases (gnomAD v3 and HelixMTdb) that provide both homoplasmic and heteroplasmic frequencies from >250,000 samples collectively, mostly depleted for severe disease.[16,21] To stay in line with field standards, we selected to integrate homoplasmic frequencies into our prioritization pipeline, given there are no recommendations in the current mtDNA-specifications of the ACMG/AMP guidelines for the use or interpretation in variant classification, and that the heteroplasmic frequency of numerous pathogenic variants exceeds the standard threshold of <1:50,000.[16] We used the recently reported heteroplasmic frequencies and maximum observed HLs in reference populations to guide careful downstream clinical evaluation of candidates, with the expectation that heteroplasmic variants with a deleterious consequence on mitochondrial function are unlikely to be tolerated at high HLs in a reference population depleted of severe early-onset disease. We also prioritized rare variants based on mitochondrial constraint using recently developed metrics[22] for regional and positional constraint, with a similar rationale that variants falling in areas of mitochondrial constraint are less tolerated in humans and may play a role in disease. As mitochondrial constraint metrics are available for all mtDNA positions, our analysis could also include rRNA and non-coding variants that are otherwise challenging to interpret due to the absence of computational prediction tools for these genomic regions. In our analysis, a number of high priority protein-coding candidates were supported by mitochondrial constraint data, as well as the somatic mtDNA variant likely driving the CSA phenotype of our *POLG* proband. At this time, all of our prioritized rRNA and non-coding variants in constrained regions were not of high enough clinical interest, based on the proband's phenotype, to pursue further.

There are a number of limitations to our study. First, the primary source of DNA for sequencing in our rare disease cohort is blood, where variant HL is typically lower than in disease-affected tissues and can further decrease over time due to rapid replication.[44] Therefore, although mtDNA variants can be detected in the blood in the majority of patients,[45] in particular during childhood, it is not the optimal source of DNA for MD diagnosis. The age at DNA sample collection was not available for our cohort to further understand the impact of this limitation on our analysis. From blood we cannot conclusively rule out an MD in our undiagnosed families. This underpinned our decision to include RNA sequencing data from probands, when available. Our RNA sequencing data are

mostly from fibroblasts or muscle tissue, offering the opportunity to capture mtDNA variants in a second tissue and potentially at a higher HL, as well as to increase the likelihood to detect large mtDNA deletions that are mostly isolated to muscle.[7] In two probands (one known diagnosis, one candidate) RNA sequencing supported the presence of the variant in a second tissue. Second, in most cases, we were unable to functionally validate candidates by gold standard methods (e.g., single fiber analysis or cybrids) due to unavailability of patient-derived tissues. Gene editing is theoretically possible for a subset of the variant types (C>T or A>G transitions), yet is highly specialized, time consuming, and challenging to pursue.[46] We, therefore, hope that, by sharing these candidate variants, we may connect with additional affected families in the future to build evidence toward pathogenic designation.

In summary, our analysis pipeline prioritized a mtDNA variant for clinical evaluation in approximately 1 per 10 probands, adding minimal additional analytical burden to nuclear genome analysis. This gave the opportunity to capture diagnostic mtDNA variants in patients who did not necessarily have a high enough clinical suspicion of MD to prompt targeted mtDNA sequencing. In our hands, mtDNA analysis resulted in the diagnosis for 0.2% (1 in 500) of undiagnosed families with diverse rare disease phenotypes, as well as the identification of additional promising candidates.

Data and code availability

Genomic and phenotypic data from the Broad CMG are available via dbGaP accession numbers phs003047 (GREGoR) and phs001272 (CMG). Access is managed by a data access committee designated by dbGaP and is based on intended use of the requester and allowed use of the data submitter as defined by consent codes. Submission to ClinVar is currently in progress for mtDNA variants that were interpreted as causal in this cohort (https://www.ncbi.nlm.nih.gov/clinvar/).

Acknowledgments

We thank the families who participate in these research studies and Dr. Vamsi Mootha and Dr. Melissa Walker for advice on mtDNA candidate evaluation. This work was supported by the National Institutes of Health (NIH) National Human Genome Research Institute (NHGRI) GREGoR Program (U01HG011758, U01HG011755, U01HG011762, U01HG011745, U01HG011744, and U24HG011746), as well as NHGRI grants UM1HG008900 (with additional support from the National Eye Institute, and the National Heart, Lung and Blood Institute [NHLBI]), and R01HG009141, National Institute of Diabetes and Digestive and Kidney Diseases (NIDDK) RC2DK122533, and in part by the Chan Zuckerberg Initiative Donor-Advised Fund at the Silicon Valley Community Foundation (grants 2019-199278, 2020-224274, and 2022-316726). S.L.S. was supported by a Manton Center for Orphan Disease Research fellowship at Boston Children's Hospital. V.S.G. was supported by the National Institute of Arthritis and Musculoskeletal and Skin Diseases grant K23AR083505. V.G.S. is supported by the Howard Hughes Medical Institute, the Alex's Lemonade Stand Foundation, and NIH grants from the National Cancer Institute (R01CA265726, R01CA292941, R33CA278393), NIDDK (R01DK103794), and NHLBI (R01HL146500). K.M.B. and E.A.P. were supported by National Eye Institute [R01EY012910 (E.A.P.), R01EY035717 (K.M. B.), and P30EY014104 (MEEI core support)]. L.G., T.Y.T., and S. M.W. acknowledge financial support from the Royal Children's Hospital Foundation, Murdoch Children's Research Institute, and the Harbig Foundation. D.R.T. and A.G.C. acknowledge support from the Australian National Health and Medical Research Council (GNT1164479 and GNT1155244) and the Mito Foundation. The research conducted at the Murdoch Children's Research Institute was supported by the Victorian Government's Operational Infrastructure Support Program. The content is solely the responsibility of the authors and does not necessarily represent the official views of the funding agencies.

Declaration of interests

A.O'D.-L. was a paid consultant to Tome Biosciences, Ono Pharma USA, Addition Therapeutics, has received research funding from Pacific Biosciences, and is on the American Journal of Human Genetics Editorial Board (unpaid). H.L.R. has received rare-disease research funding from Microsoft. V.G.S. serves as an advisor to Ensoma.

Supplemental information

Supplemental information can be found online at https://doi.org/10.1016/j.xhgg.2025.100441.

Web resources

MITOMAP: https://www.mitomap.org/MITOMAP
ClinVar: https://www.ncbi.nlm.nih.gov/clinvar/

Received: January 29, 2025
Accepted: April 13, 2025

References

1. Stenton, S.L., and Prokisch, H. (2020). Genetics of mitochondrial diseases: Identifying mutations to help diagnosis. EBioMedicine *56*, 102784.
2. Gorman, G.S., Chinnery, P.F., DiMauro, S., Hirano, M., Koga, Y., McFarland, R., Suomalainen, A., Thorburn, D.R., Zeviani, M., and Turnbull, D.M. (2016). Mitochondrial diseases. Nat. Rev. Dis. Primers *2*, 16080.
3. Frazier, A.E., Thorburn, D.R., and Compton, A.G. (2019). Mitochondrial energy generation disorders: genes, mechanisms, and clues to pathology. J. Biol. Chem. *294*, 5386–5395.
4. Barca, E., Long, Y., Cooley, V., Schoenaker, R., Emmanuele, V., DiMauro, S., Cohen, B.H., Karaa, A., Vladutiu, G.D., Haas, R., et al. (2020). Mitochondrial diseases in North America: An analysis of the NAMDC Registry. Neurol. Genet. *6*, e402.
5. Gorman, G.S., Schaefer, A.M., Ng, Y., Gomez, N., Blakely, E. L., Alston, C.L., Feeney, C., Horvath, R., Yu-Wai-Man, P., Chinnery, P.F., et al. (2015). Prevalence of nuclear and

mitochondrial DNA mutations related to adult mitochondrial disease. Ann. Neurol. *77*, 753–759.
6. Lott, M.T., Leipzig, J.N., Derbeneva, O., Xie, H.M., Chalkia, D., Sarmady, M., Procaccio, V., and Wallace, D.C. (2013). mtDNA Variation and Analysis Using Mitomap and Mitomaster. Curr. Protoc. Bioinformatics *44*. 1.23.1-26.
7. Stewart, J.B., and Chinnery, P.F. (2015). The dynamics of mitochondrial DNA heteroplasmy: implications for human health and disease. Nat. Rev. Genet. *16*, 530–542.
8. Longo, N. (2003). Mitochondrial encephalopathy. Neurol. Clin. *21*, 817–831.
9. Yu-Wai-Man, P., Votruba, M., Burté, F., La Morgia, C., Barboni, P., and Carelli, V. (2016). A neurodegenerative perspective on mitochondrial optic neuropathies. Acta Neuropathol. *132*, 789–806.
10. Stenton, S.L., Shimura, M., Piekutowska-Abramczuk, D., Freisinger, P., Distelmaier, F., Mayr, J.A., Makowski, C., Büchner, B., Alhaddad, B., Alston, C.L., et al. (2021). Diagnosing pediatric mitochondrial disease: lessons from 2,000 exomes. Preprint at: medRxiv. 2021.06.21.21259171. https://doi.org/10.1101/2021.06.21.21259171
11. Gorman, G.S., McFarland, R., Stewart, J., Feeney, C., and Turnbull, D.M. (2018). Mitochondrial donation: from test tube to clinic. Lancet *392*, 1191–1192.
12. Wortmann, S.B., Koolen, D.A., Smeitink, J.A., van den Heuvel, L., and Rodenburg, R.J. (2015). Whole exome sequencing of suspected mitochondrial patients in clinical practice. J. Inherit. Metab. Dis. *38*, 437–443.
13. Poole, O.V., Pizzamiglio, C., Murphy, D., Falabella, M., Macken, W.L., Bugiardini, E., Woodward, C.E., Labrum, R., Efthymiou, S., Salpietro, V., et al. (2021). Mitochondrial DNA Analysis from Exome Sequencing Data Improves Diagnostic Yield in Neurological Diseases. Ann. Neurol. *89*, 1240–1247.
14. Wagner, M., Berutti, R., Lorenz-Depiereux, B., Graf, E., Eckstein, G., Mayr, J.A., Meitinger, T., Ahting, U., Prokisch, H., Strom, T.M., and Wortmann, S.B. (2019). Mitochondrial DNA mutation analysis from exome sequencing-A more holistic approach in diagnostics of suspected mitochondrial disease. J. Inherit. Metab. Dis. *42*, 909–917.
15. Schon, K.R., Horvath, R., Wei, W., Calabrese, C., Tucci, A., Ibañez, K., Ratnaike, T., Pitceathly, R.D.S., Bugiardini, E., Quinlivan, R., et al. (2021). Use of whole genome sequencing to determine genetic basis of suspected mitochondrial disorders: cohort study. BMJ *375*, e066288.
16. Laricchia, K.M., Lake, N.J., Watts, N.A., Shand, M., Haessly, A., Gauthier, L., Benjamin, D., Banks, E., Soto, J., Garimella, K., et al. (2022). Mitochondrial DNA variation across 56,434 individuals in gnomAD. Genome Res. *32*, 569–582.
17. Calabrese, C., Simone, D., Diroma, M.A., Santorsola, M., Guttà, C., Gasparre, G., Picardi, E., Pesole, G., and Attimonelli, M. (2014). MToolBox: a highly automated pipeline for heteroplasmy annotation and prioritization analysis of human mitochondrial variants in high-throughput sequencing. Bioinformatics *30*, 3115–3117.
18. Basu, S., Xie, X., Uhler, J.P., Hedberg-Oldfors, C., Milenkovic, D., Baris, O.R., Kimoloi, S., Matic, S., Stewart, J.B., Larsson, N. G., et al. (2020). Accurate mapping of mitochondrial DNA deletions and duplications using deep sequencing. PLoS Genet. *16*, e1009242.
19. Falk, M.J., Pierce, E.A., Consugar, M., Xie, M.H., Guadalupe, M., Hardy, O., Rappaport, E.F., Wallace, D.C., LeProust, E., and Gai, X. (2012). Mitochondrial disease genetic diagnostics: optimized whole-exome analysis for all MitoCarta nuclear genes and the mitochondrial genome. Discov. Med. *14*, 389–399.
20. Riley, L.G., Cowley, M.J., Gayevskiy, V., Minoche, A.E., Puttick, C., Thorburn, D.R., Rius, R., Compton, A.G., Menezes, M.J., Bhattacharya, K., et al. (2020). The diagnostic utility of genome sequencing in a pediatric cohort with suspected mitochondrial disease. Genet. Med. *22*, 1254–1261.
21. Bolze, A., Mendez, F., White, S., Tanudjaja, F., Isaksson, M., Jiang, R., Rossi, A.D., Cirulli, E.T., Rashkin, M., Metcalf, W. J., et al. (2020). A catalog of homoplasmic and heteroplasmic mitochondrial DNA variants in humans. Preprint at: bioRxiv, 798264. https://doi.org/10.1101/798264
22. Lake, N.J., Ma, K., Liu, W., Battle, S.L., Laricchia, K.M., Tiao, G., Puiu, D., Ng, K.K., Cohen, J., Compton, A.G., et al. (2024). Quantifying constraint in the human mitochondrial genome. Nature *635*, 390–397.
23. Landrum, M.J., Lee, J.M., Benson, M., Brown, G., Chao, C., Chitipiralla, S., Gu, B., Hart, J., Hoffman, D., Hoover, J., et al. (2016). ClinVar: public archive of interpretations of clinically relevant variants. Nucleic Acids Res. *44*, D862–D868.
24. Bianco, S.D., Parca, L., Petrizzelli, F., Biagini, T., Giovannetti, A., Liorni, N., Napoli, A., Carella, M., Procaccio, V., Lott, M. T., et al. (2023). APOGEE 2: multi-layer machine-learning model for the interpretable prediction of mitochondrial missense variants. Nat. Commun. *14*, 5058.
25. Preste, R., Vitale, O., Clima, R., Gasparre, G., and Attimonelli, M. (2019). HmtVar: a new resource for human mitochondrial variations and pathogenicity data. Nucleic Acids Res. *47*, D1202–D1210.
26. Sonney, S., Leipzig, J., Lott, M.T., Zhang, S., Procaccio, V., Wallace, D.C., and Sondheimer, N. (2017). Predicting the pathogenicity of novel variants in mitochondrial tRNA with MitoTIP. PLoS Comput. Biol. *13*, e1005867.
27. Niroula, A., and Vihinen, M. (2016). PON-mt-tRNA: a multifactorial probability-based method for classification of mitochondrial tRNA variations. Nucleic Acids Res. *44*, 2020–2027.
28. McCormick, E.M., Lott, M.T., Dulik, M.C., Shen, L., Attimonelli, M., Vitale, O., Karaa, A., Bai, R., Pineda-Alvarez, D.E., Singh, L.N., et al. (2020). Specifications of the ACMG/AMP standards and guidelines for mitochondrial DNA variant interpretation. Hum. Mutat. *41*, 2028–2057.
29. Morava, E., van den Heuvel, L., Hol, F., de Vries, M.C., Hogeveen, M., Rodenburg, R.J., and Smeitink, J.A.M. (2006). Mitochondrial disease criteria: diagnostic applications in children. Neurology *67*, 1823–1826.
30. Slomovic, S., Laufer, D., Geiger, D., and Schuster, G. (2005). Polyadenylation and degradation of human mitochondrial RNA: the prokaryotic past leaves its mark. Mol. Cell Biol. *25*, 6427–6435.
31. Pickett, S.J., Grady, J.P., Ng, Y.S., Gorman, G.S., Schaefer, A. M., Wilson, I.J., Cordell, H.J., Turnbull, D.M., Taylor, R.W., and McFarland, R. (2018). Phenotypic heterogeneity in m.3243A>G mitochondrial disease: The role of nuclear factors. Ann. Clin. Transl. Neurol. *5*, 333–345.
32. Ratnaike, T.E., Greene, D., Wei, W., Sanchis-Juan, A., Schon, K.R., van den Ameele, J., Raymond, L., Horvath, R., Turro, E., and Chinnery, P.F. (2021). MitoPhen database: a human phenotype ontology-based approach to identify mitochondrial DNA diseases. Nucleic Acids Res. *49*, 9686–9695.

33. Huang, L.S., Cobessi, D., Tung, E.Y., and Berry, E.A. (2005). Binding of the respiratory chain inhibitor antimycin to the mitochondrial bc1 complex: a new crystal structure reveals an altered intramolecular hydrogen-bonding pattern. J. Mol. Biol. *351*, 573–597.
34. Blakely, E.L., Yarham, J.W., Alston, C.L., Craig, K., Poulton, J., Brierley, C., Park, S.M., Dean, A., Xuereb, J.H., Anderson, K.N., et al. (2013). Pathogenic mitochondrial tRNA point mutations: nine novel mutations affirm their importance as a cause of mitochondrial disease. Hum. Mutat. *34*, 1260–1268.
35. Wang, J., Balciuniene, J., Diaz-Miranda, M.A., McCormick, E. M., Aref-Eshghi, E., Muir, A.M., Cao, K., Troiani, J., Moseley, A., Fan, Z., et al. (2022). Advanced approach for comprehensive mtDNA genome testing in mitochondrial disease. Mol. Genet. Metab. *135*, 93–101.
36. Longley, M.J., Ropp, P.A., Lim, S.E., and Copeland, W.C. (1998). Characterization of the native and recombinant catalytic subunit of human DNA polymerase gamma: identification of residues critical for exonuclease activity and dideoxynucleotide sensitivity. Biochemistry *37*, 10529–10539.
37. Wanrooij, S., Goffart, S., Pohjoismäki, J.L.O., Yasukawa, T., and Spelbrink, J.N. (2007). Expression of catalytic mutants of the mtDNA helicase Twinkle and polymerase POLG causes distinct replication stalling phenotypes. Nucleic Acids Res. *35*, 3238–3251.
38. Maclaine, K.D., Stebbings, K.A., Llano, D.A., and Havird, J.C. (2021). The mtDNA mutation spectrum in the PolG mutator mouse reveals germline and somatic selection. BMC Genom. Data *22*, 52.
39. Wong, L.J.C., Naviaux, R.K., Brunetti-Pierri, N., Zhang, Q., Schmitt, E.S., Truong, C., Milone, M., Cohen, B.H., Wical, B., Ganesh, J., et al. (2008). Molecular and clinical genetics of mitochondrial diseases due to POLG mutations. Hum. Mutat. *29*, E150–E172.
40. Ducamp, S., and Fleming, M.D. (2019). The molecular genetics of sideroblastic anemia. Blood *133*, 59–69.
41. Pontikos, N., Arno, G., Jurkute, N., Schiff, E., Ba-Abbad, R., Malka, S., Gimenez, A., Georgiou, M., Wright, G., Armengol, M., et al. (2020). Genetic Basis of Inherited Retinal Disease in a Molecularly Characterized Cohort of More Than 3000 Families from the United Kingdom. Ophthalmology *127*, 1384–1394.
42. Sallevelt, S.C.E.H., de Die-Smulders, C.E.M., Hendrickx, A. T.M., Hellebrekers, D.M.E.I., de Coo, I.F.M., Alston, C.L., Knowles, C., Taylor, R.W., McFarland, R., and Smeets, H. J.M. (2017). De novo mtDNA point mutations are common and have a low recurrence risk. J. Med. Genet. *54*, 73–83.
43. Falk, M.J., Shen, L., Gonzalez, M., Leipzig, J., Lott, M.T., Stassen, A.P.M., Diroma, M.A., Navarro-Gomez, D., Yeske, P., Bai, R., et al. (2015). Mitochondrial Disease Sequence Data Resource (MSeqDR): a global grass-roots consortium to facilitate deposition, curation, annotation, and integrated analysis of genomic data for the mitochondrial disease clinical and research communities. Mol. Genet. Metab. *114*, 388–396.
44. Grady, J.P., Pickett, S.J., Ng, Y.S., Alston, C.L., Blakely, E.L., Hardy, S.A., Feeney, C.L., Bright, A.A., Schaefer, A.M., Gorman, G.S., et al. (2018). mtDNA heteroplasmy level and copy number indicate disease burden in m.3243A>G mitochondrial disease. EMBO Mol. Med. *10*, e8262. https://doi.org/10.15252/emmm.201708262.
45. Raymond, F.L., Horvath, R., and Chinnery, P.F. (2018). First-line genomic diagnosis of mitochondrial disorders. Nat. Rev. Genet. *19*, 399–400.
46. Shoop, W.K., Bacman, S.R., Barrera-Paez, J.D., and Moraes, C. T. (2023). Mitochondrial gene editing. Nat. Rev. Methods Primers *3*, 19.

HGGA, Volume *6*

Supplemental information

Mitochondrial DNA variant detection in over 6,500 rare disease families by the systematic analysis of exome and genome sequencing data resolves undiagnosed cases

Sarah L. Stenton, Kristen Laricchia, Nicole J. Lake, Sushma Chaluvadi, Vijay Ganesh, Stephanie DiTroia, Ikeoluwa Osei-Owusu, Lynn Pais, Emily O'Heir, Christina Austin-Tse, Melanie O'Leary, Mayada Abu Shanap, Chelsea Barrows, Seth Berger, Carsten G. Bönnemann, Kinga M. Bujakowska, Dean R. Campagna, Alison G. Compton, Sandra Donkervoort, Mark D. Fleming, Lyndon Gallacher, Joseph G. Gleeson, Goknur Haliloglu, Eric A. Pierce, Emily M. Place, Vijay G. Sankaran, Akiko Shimamura, Zornitza Stark, Tiong Yang Tan, David R. Thorburn, Susan M. White, Maha S. Zaki, Genomics Research to Elucidate the Genetics of Rare diseases (GREGoR) Consortium, Eric Vilain, Monkol Lek, Heidi L. Rehm, and Anne O'Donnell-Luria

Supplemental Materials

1. Supplemental Results (page 2)
2. Supplemental Figures and Legends (pages 3-6)
3. Supplemental Tables (page 7)
4. Supplemental Methods (pages 8-10)
5. Supplemental References (pages 11-12)

1. Supplemental results

Comparison of variant calls from the "gnomad-mitochondria" pipeline and the MToolBox pipeline for 24 GS samples. The "gnomad-mitochondria" pipeline recalled 81% (540/669) of variants detected at ≥1% HL by the MToolBox pipeline and 99% (522/526) detected at ≥5% (analysis excluded difficult to sequence regions) (**Figure S3A**). For variants detected by both pipelines (540 variants), correlation in HL was high (R=0.99, p-value<0.0001, Pearson Correlation Coefficient) (**Figure S3B**) and the mean difference in HL per variant was 0.06%. The pipelines agreed on the top level haplogroup for all 24 samples.

Comparison of variant calls from the 100 paired GS to RNA-sequencing samples. Applying the MToolBox pipeline to RNA-sequencing data recalled 92% (1,295/1,408) of variants detected at ≥1% HL in the corresponding GS data and 97% (1,283/1,323) detected at ≥5% (analysis excluded difficult to sequence regions, indels, and non-coding regions) (**Figure S4A**). For variants detected by both pipelines (1,295 variants), correlation in HL was high (R=0.99, p-value<0.0001, Pearson Correlation Coefficient) (**Figure S4A**) and the mean difference in HL per variant was 1.2%. The data types agreed on the top level haplogroup for 98/100 (98%) samples. The remaining two RNA-sequencing samples had missing values for top level haplogroup.

2. Supplemental Figures

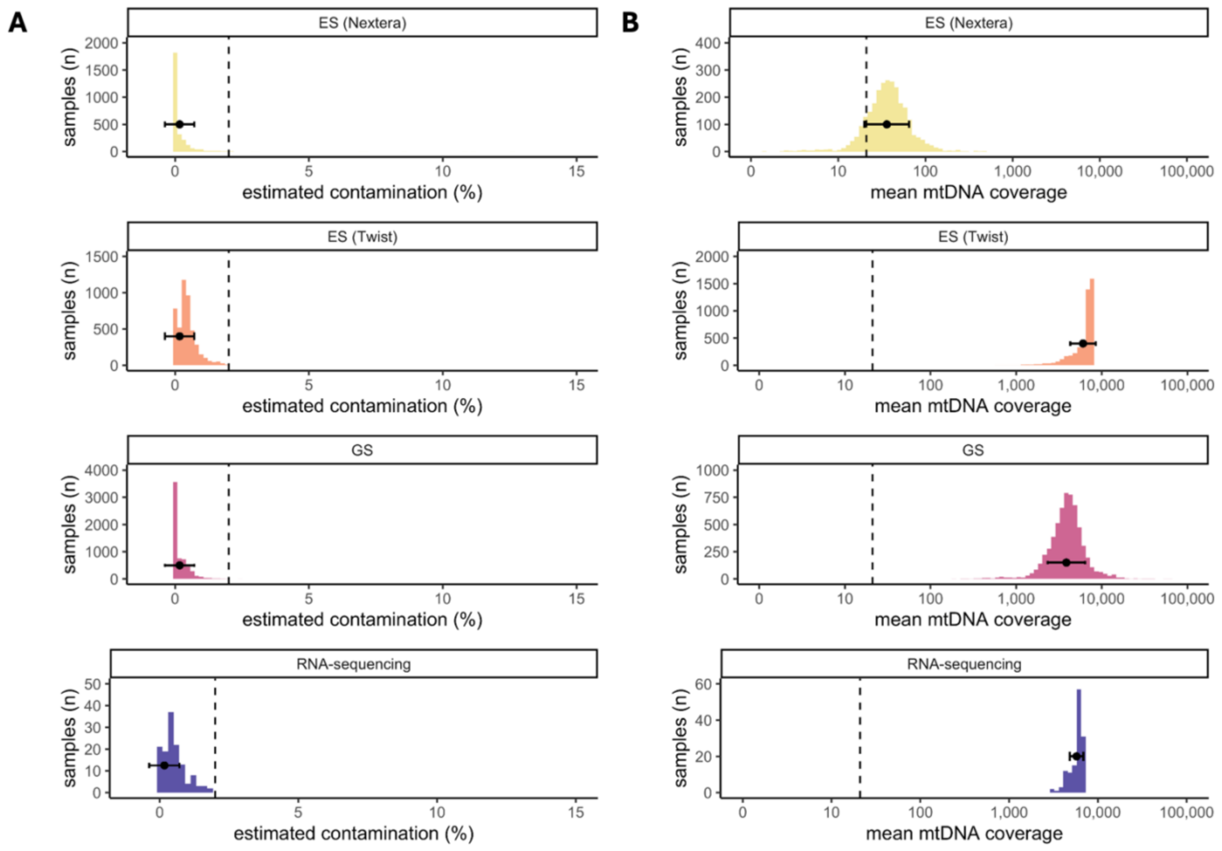

Figure S1. Sample level QC metrics by data type. A. Estimated contamination (%). Samples with ≥2% contamination with high heteroplasmic haplogroup defining variants (dashed line) were excluded from the study cohort. **B.** Mean mtDNA coverage. Samples with a mean mtDNA coverage <20X (dashed line) were excluded from the study cohort. Data are displayed as histograms with corresponding mean and standard deviation.

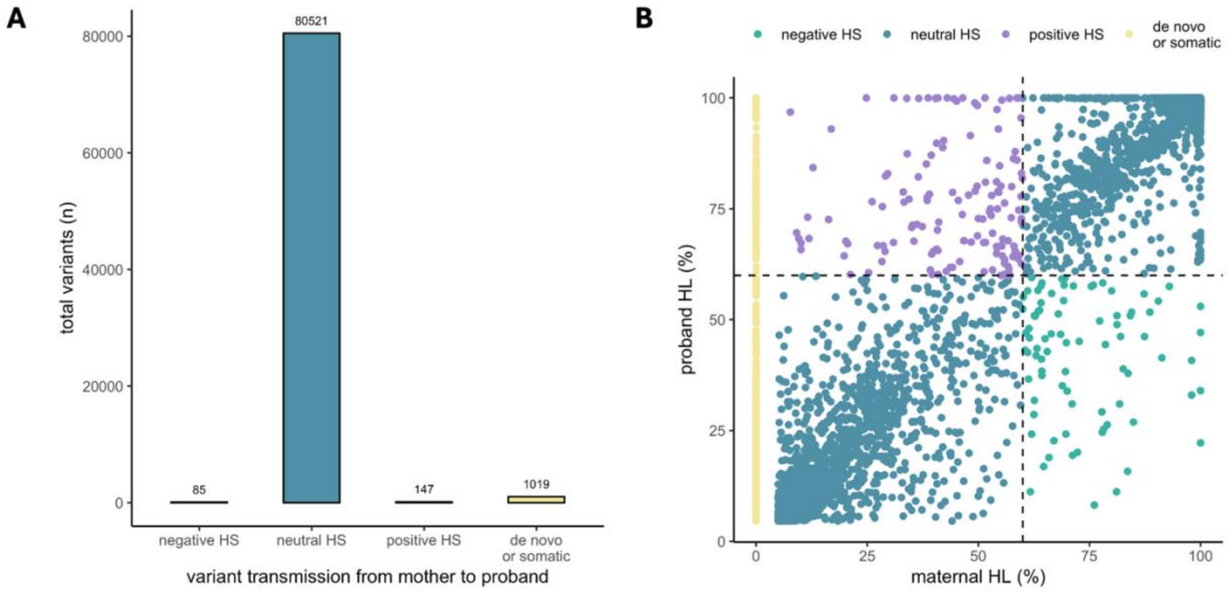

Figure S2. Comparison of variant heteroplasmic level in paired maternal and proband samples. A. Total number of variants in available paired mother-proband data by variant transmission to the proband. **B.** Comparison of the HL of variants detected in the proband with the HL in the maternal sample, dashed lines at 60% HL indicate the typical threshold that should be exceeded to cause disease. HS, heteroplasmic shift; HL, heteroplasmy level.

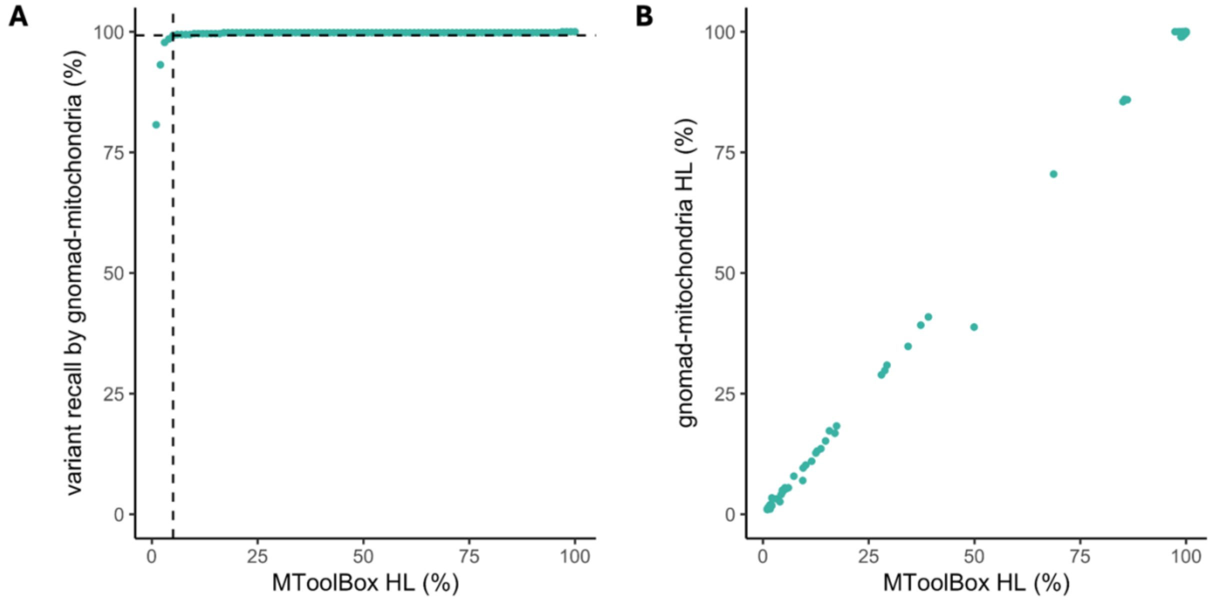

Figure S3. Comparison of variant calls from the “gnomad-mitochondria” pipeline and the MToolBox pipeline. **A.** Variants detected by the MToolBox pipeline displayed by heteroplasmy level with the corresponding recall by gnomad-mitochondria. **B.** Correlation between the heteroplasmy level of the variants detected by both pipelines.

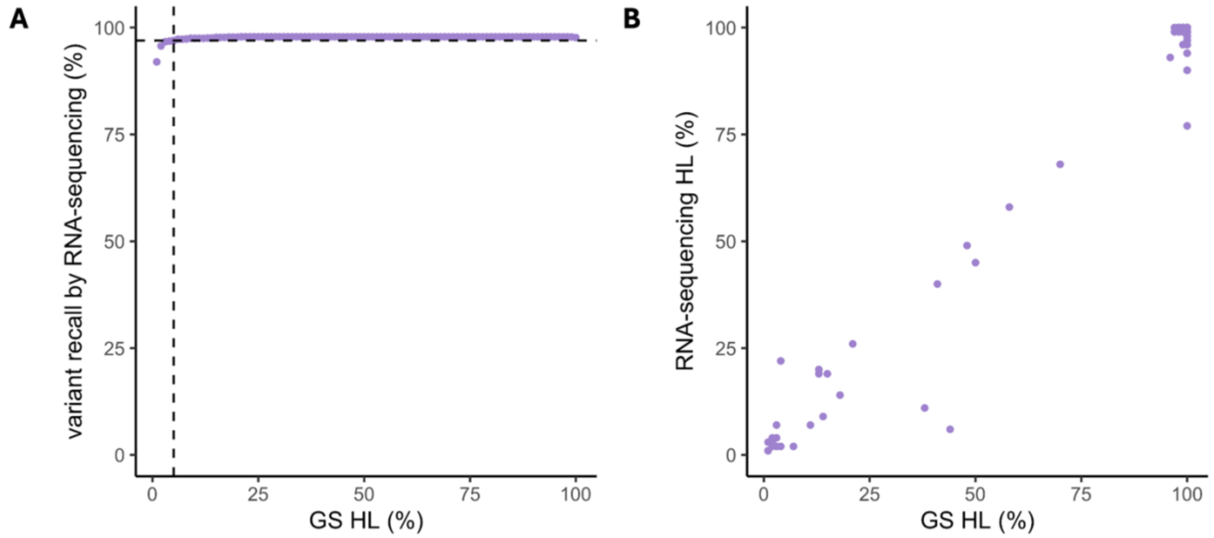

Figure S4. Comparison of variant calls from the 100 paired GS to RNA-sequencing samples. **A.** Variants detected in the GS pipeline displayed by heteroplasmy level with the corresponding recall by RNA-sequencing. **B.** Correlation between the heteroplasmy level of the variants detected by both GS and RNA-sequencing.

3. **Supplemental Tables**

Table S1. MITOMAP confirmed and ClinVar P/LP variants (separate file).

**Data type**	**Proband samples** (n)	**Variant count per sample** (mean)	
		**Homoplasmic** (≥95% HL)	**Heteroplasmic** (0.05≤ HL <0.95)
ES (Nextera)	1,647	7.7	1.5
ES (Twist)	2,276	30	2.7
GS	2,759	27	1.9
RNA-sequencing*	124	13	2.0

Table S2. Counts of high-quality variants per sample and per proband for analysis by data type. *RNA-sequencing analysis only includes SNVs in protein-coding regions.

4. Supplemental Methods

mtDNA variant calling and haplogroup determination. For genome sequencing (GS), the mitochondria mode of GATK-Mutect2 was run in Terra using the "gnomad-mitochondria" WDL file available on GitHub (https://github.com/broadinstitute/gnomad-mitochondria) for SNV and small indel calling plus haplogroup determination as previously described.[1] Default parameters were used with the exception of "vaf_filter_threshold", which was set to 0.01 to flag all variants at HL<1% as "low_allele_frac", considered as homoplasmic reference. Each GS sample-level per base coverage and VCF WDL output file was analyzed downstream in Hail version 0.2.105.[2] Each GS sample-level mtDNA aligned BAM WDL output file was used as input to the MitoSAlt pipeline for large (≥50bp) mtDNA deletion calling.[3]

For exome sequencing (ES) and RNA-sequencing, the MToolBox pipeline (https://github.com/mitoNGS/MToolBox) was used for SNV and small indel calling plus haplogroup determination as previously described.[4] Default installation of MToolBox was used (install.sh). In the MToolBox config file the following mandatory parameters were set: "input_type=fastq", "ref=RCRS". All other parameters remained as default. CRAM/BAM files were converted to FASTQ files using samtools prior to running the MToolBox pipeline. Each ES/RNA-seq sample-level haplogroup annotation, per base coverage, and VCF output file was analyzed downstream in Hail version 0.2.105.[2] Each ES/RNA-seq sample-level FASTQ file containing mtDNA mapped reads was used as input to the MitoSAlt pipeline for large (≥50bp) mtDNA deletion calling.[3]

For all data types, large mtDNA deletions were identified with MitoSAlt (https://sourceforge.net/projects/mitosalt/).[3] Default parameters were used, with "dna = yes" for ES/GS and "dna = no" for RNA-seq, plus "enriched = yes" for all data types in the config file. These parameters indicate the source of the input data and that the reads are enriched for mtDNA mapped reads given that all input data were previously mapped to the mtDNA and depleted of nuclear reads. Each sample's MitoSAlt tab-delimited output file of deletion calls was analyzed downstream in R version 4.1.1.

As the "gnomad-mitochondria" pipeline had not previously been applied for rare disease diagnostic purposes and as the MToolBox pipeline had not previously been

applied to RNA-seq data, we ran analyses of variant recall comparing "gnomad-mitochondria" to MToolBox in 24 GS samples and comparing RNA-seq to GS in 100 paired samples (see **Supplemental Results**).

mtDNA variant annotation. Flags were added to mark low quality variant calls that were likely false positives, as follows: i) variants at positions with coverage depth <20X were flagged as "low coverage" in the respective sample in order to only retain samples expected to have an equal or lower sequencing error rate to Sanger sequencing;[5] ii) variants in reported difficult to sequence regions (M-300:316, M-513:525, and M-16182:16194) were flagged as "artifact prone site"; iii) indel stacks, defined as indels at positions that are multi-allelic across samples in our cohort and/or gnomAD v3, were flagged as "indel stack", and iv) variants with a HL below 5% were flagged as "low heteroplasmy" due to high risk of being enriched for NUMT-derived signals. In the RNA-sequencing data, all indels variants and all SNV variants outside of the protein-coding regions were flagged for removal.

All variants were annotated with the respective gene name and gene function (protein-coding, tRNA, or rRNA) or as non-coding. Variant consequence was annotated with the Ensembl Variant Effect Predictor (VEP).[6] Variants were flagged as haplogroup defining according to Phylotree and were downloaded from the "gnomad-mitochondria" GitHub resources folder ("rCRS-centered_phylo_vars_final_update.txt"). Homoplasmic and heteroplasmic allele frequencies reported in gnomAD v3[1] and HelixMTdb (https://www.helix.com/pages/mitochondrial-variant-database)[7] reference population databases were annotated in addition to the maximum observed HL. The following computational predictions were annotated based on gene function and/or variant function: i) protein-coding and tRNA variants with HmtVar scores using the HmtVar API (https://www.hmtvar.uniba.it/);[8] ii) missense variants with APOGEE2 scores, downloaded from MitImpact (https://mitimpact.css-mendel.it/);[9,10] iii) tRNA variants with MitoTIP scores downloaded from MITOMAP (https://www.mitomap.org/foswiki/bin/view/MITOMAP/MitoTipInfo),[11,12] and PON-mt-tRNA scores downloaded from (http://structure.bmc.lu.se/PON-mt-tRNA/datasets.html/).[13] Mitochondrial constraint metrics - regional constraint and

mitochondrial local constraint (MLC) scores[14] - were annotated. When data were available from the mother of the proband, the transmission of the variant was annotated as follows: i) variants detected in both the mother and proband at ≥1% HL were considered transmitted and annotated as either transmitted with “clinically significant positive heteroplasmic shift” (mother HL<60%, proband HL≥60%), “neutral heteroplasmic shift” (both mother and proband HL≥60% or HL<60%), or “clinically significant negative heteroplasmic shift” (proband HL<60%, mother HL≥0.6) in the proband; ii) variants present in the proband and not detected in the mother were annotated as “presumed *de novo* or somatic” in the proband.

5. Supplemental References

1. Laricchia KM, Lake NJ, Watts NA, et al. Mitochondrial DNA variation across 56,434 individuals in gnomAD. *Genome Res*. 2022;32(3):569-582.

2. Hail Team. Hail 0.2.105-acd89e80c345. GitHub.

3. Basu S, Xie X, Uhler JP, et al. Accurate mapping of mitochondrial DNA deletions and duplications using deep sequencing. *PLoS Genet*. 2020;16(12):e1009242.

4. Calabrese C, Simone D, Diroma MA, et al. MToolBox: a highly automated pipeline for heteroplasmy annotation and prioritization analysis of human mitochondrial variants in high-throughput sequencing. *Bioinformatics*. 2014;30(21):3115-3117.

5. Griffin HR, Pyle A, Blakely EL, et al. Accurate mitochondrial DNA sequencing using off-target reads provides a single test to identify pathogenic point mutations. *Genet Med*. 2014;16(12):962-971.

6. McLaren W, Gil L, Hunt SE, et al. The Ensembl Variant Effect Predictor. *Genome Biol*. 2016;17(1):122.

7. Bolze A, Mendez F, White S, et al. A catalog of homoplasmic and heteroplasmic mitochondrial DNA variants in humans. *bioRxiv*. Published online June 26, 2020:798264. doi:10.1101/798264

8. Preste R, Vitale O, Clima R, Gasparre G, Attimonelli M. HmtVar: a new resource for human mitochondrial variations and pathogenicity data. *Nucleic Acids Res*. 2019;47(D1):D1202-D1210.

9. Castellana S, Fusilli C, Mazzoccoli G, et al. High-confidence assessment of functional impact of human mitochondrial non-synonymous genome variations by APOGEE. *PLoS Comput Biol*. 2017;13(6):e1005628.

10. Bianco SD, Parca L, Petrizzelli F, et al. APOGEE 2: multi-layer machine-learning model for the interpretable prediction of mitochondrial missense variants. *Nat Commun*. 2023;14(1):5058.

11. Sonney S, Leipzig J, Lott MT, et al. Predicting the pathogenicity of novel variants in mitochondrial tRNA with MitoTIP. *PLoS Comput Biol*. 2017;13(12):e1005867.

12. Lott MT, Leipzig JN, Derbeneva O, et al. mtDNA Variation and Analysis Using Mitomap and Mitomaster. *Curr Protoc Bioinformatics*. 2013;44(123):1.23.1-26.

13. Niroula A, Vihinen M. PON-mt-tRNA: a multifactorial probability-based method for classification of mitochondrial tRNA variations. *Nucleic Acids Res*. 2016;44(5):2020-2027.

14. Lake NJ, Ma K, Liu W, et al. Quantifying constraint in the human mitochondrial genome. *Nature*. Published online October 16, 2024:1-8.
